# Characteristics of Medical School Deans and University Hospital Directors in Japan

**DOI:** 10.1001/jamanetworkopen.2023.51526

**Published:** 2024-01-11

**Authors:** Takashi Watari, Ashwin Gupta, Maika Hayashi, Kotoko Mizuno, Yasuhisa Nakano, Yasuharu Tokuda, Kota Sakaguchi

**Affiliations:** 1 Department of Medicine, University of Michigan Medical School, Ann Arbor; 2 General Medicine Center, Shimane University, Shimane, Japan; 3 Medicine Service, VA Ann Arbor Healthcare System, Ann Arbor, Michigan; 4 Faculty of Medicine, Shimane University, Shimane, Japan; 5 Muribushi Okinawa Clinical Training Center, Okinawa, Japan

## Abstract

This cross-sectional study investigates characteristics, including sex, postgraduate experience, and specialty, of medical school deans and university hospital directors in Japan.

## Introduction

Medical school deans and university hospital directors are pivotal high-ranking leaders in health care organizations, exerting profound influence on the clinical, research, educational, and managerial directions of the organization, medical discipline, and functioning of the medical-social health system.^1,2^ Selecting high-level leaders in academic medicine who are from diverse backgrounds in an equitable and transparent way is imperative^1,2,3^; however, background characteristics of university hospital directors and medical school deans in Japan remain largely unexplored.

## Methods

This cross-sectional study was approved by the Institutional Review Board of Shimane University, which granted an exemption for informed consent because we exclusively relied on publicly available information. We adhered to the STROBE reporting guideline.

The study identified medical school deans and hospital directors from 82 universities authorized and listed by the Ministry of Education, Culture, Sports, Science, and Technology as of June 1, 2022. Characteristics, such as sex, years since medical school graduation, university association, specialization area, research field, and doctoral degrees, were obtained from university and Ministry of Health, Labor, and Welfare Medical Policy Bureau websites (eMethods 1 in Supplement 1). We used general descriptive statistics to compute the number, percentage, median, and IQR for each category and χ^2^ tests to compare nominal variables. Statistical analyses were performed using Stata statistical software version 17.0 (StataCorp). All tests were 2-tailed, and a *P* < .05 indicated statistical significance.

## Results

All 164 leaders from 82 universities (82 medical school deans and 82 hospital directors) were males and had graduated from Japanese medical schools. Each had a PhD degree, of which 98.8% were in the field of basic experimental laboratory medicine (Table 1). The median (IQR) postgraduate experience was 38 (36-40) years. Deans and directors most often specialized in internal medicine (37 individuals [22.6%]), followed by surgery (30 individuals [18.3%]), basic medicine (14 individuals [8.5%]), and urology (10 individuals [6.1%]). Among medical school deans, the greatest proportion were basic science and pathology faculty members (22 individuals [26.8%]), whereas most hospital directors were surgical faculty members (51 individuals [62.2%]). Comparing the 7 former imperial universities in Japan (established before World War II and considered Japan’s most prestigious universities) and the historical top 17 universities (14 public and 3 private universities) with other universities (eMethods 2 in Supplement 1) highlighted that leaders were more likely to be alumni of the school that they currently led and graduates of former imperial universities. Greater proportions of leaders who were alumni or imperial university graduates were also noted when comparing public with private schools (Table 2).

**Table 1.** Background Characteristics of Study Population

Characteristic^a^	Individuals, No. (%)			*P* value
	Total (N = 164)	Dean (n = 82)	Director (n = 82)	
Sex				
Male	164 (100)	82 (100)	82 (100)	NA
Female	0	0	0	
Postgraduate year, median (IQR)	38 (36-49)	39 (37-41)	38 (36-40)	.55
Specialty category				
Clinical	149 (90.9)	67 (81.7)	82 (100)	<.001
Basic science	12 (7.3)	12 (14.6)	0	<.001
Social and public health	3 (1.8)	3 (3.7)	0	.25
Degree (PhD)	164 (100)	82 (100)	82 (100)	NA
Experimental basic medicine	162 (98.8)	80 (97.6)	82 (100)	.50
Clinical research	0	0	0	NA
Social and public health	2 (1.2)	2 (2.4)	0	.50
Graduated from public medical school	123 (75)	61 (74/3_	62 (75.6)	>.99
Top 17 school (historical ranking)	49 (29.9)	26 (31.7)	23 (28.1)	.61
Alma mater^b^	90 (54.9)	48 (58.5)	42 (51.2)	.43
Specialty				
Internal medicine	37 (22.6)	19 (23.2)	18 (22)	>.99
Surgery^c^	30 (18.3)	13 (15.9)	17 (20.7)	.42
Basic, experimental medicine^d^	14 (8.5)	14 (17.1)	0	<.001
Urology^e^	10 (6.1)	1 (1.2)	9 (11)	.02
Orthopedic surgery^e^	8 (4.9)	0	8 (9.8)	.01
Obstetrics and gynecology^e^	8 (4.9)	3 (3.7)	5 (6.1)	.72
Radiology	8 (4.9)	4 (4.9)	4 (4.9)	>.99
Pathology^d^^,^^e^	8 (4.9)	8 (9.8)	0	<.001
Otolaryngology^e^	7 (4.3)	3 (3.7)	4 (4.9)	>.99
Pediatrics	5 (3.0)	3 (3.7)	2 (2.4)	>.99
Psychiatry	5 (3.0)	4 (4.9)	1 (1.2)	.37
Neurosurgery^e^	5 (3.0)	0	5 (6.1)	.06
Anesthesiology	4 (2.4)	0	4 (4.9)	.37
Ophthalmology^e^	4 (2.4)	2 (2.4)	2 (2.4)	>.99
Emergency medicine	4 (2.4)	3 (3.7)	1 (1.2)	.62
Dermatology^e^	2 (1.2)	1 (1.2)	1 (1.2)	>.99
Clinical laboratory medicine	2 (1.2)	2 (2.4)	0	.50
Public health	2 (1.2)	2 (2.4)	0	.50
General medicine	1 (0.6)	0	1 (1.2)	>.99

Abbreviation: NA, not applicable.

^a^ See eMethods 1 in Supplement 1 for details on individual data for deans and directors.

^b^ The alma mater is defined as the medical school from which the individual graduated.

^c^ The definition of surgery includes specializations, such as general, gastrointestinal, thoracic, cardiovascular, pediatric, breast, and endocrine surgery.

^d^ Basic, experimental medicine and pathology are often categorized together in Japan.

^e^ Categories of surgical faculties include surgery, urology, orthopedics, obstetrics and gynecology, neurosurgery, otolaryngology, ophthalmology, and dermatology.

**Table 2.** Differences in Characteristics by University Ranking and Funding Type

Characteristic	Leaders, No. (%)^a^		χ^2^	*P* value
	Evaluated schools	Remaining schools		
**Former imperial vs remaining 75 schools**				
Total No.	14	150	NA	NA
Graduated public medical school	14 (100)	109 (72.7)	5.10	.02
Alma mater	14 (100)	76 (50.7)	12.59	<.001
Graduated former imperial university	14 (100)	35 (23.3)	35.92	<.001
**Top 17 vs remaining 65 schools **				
Total No.	34	130	NA	NA
Graduated public medical school	27 (79.4)	96 (73.9)	0.45	.505
Alma mater	30 (88.2)	60 (46.2)	19.27	<.001
Graduated former imperial university	17 (50)	32 (24.6)	8.29	.004
**Public vs private schools**				
Total No.	102	62	NA	NA
Graduated public medical school	98 (96.1)	25 (40.3)	63.93	<.001
Alma mater	63 (61.8)	26 (42.6)	5.17	.002
Graduated former imperial university	34 (33.3)	15 (24.6)	1.54	<.001

Abbreviation: NA, not applicable.

^a^ Characteristics of leaders are compared between those at former imperial (top 7), top 17, and public schools (evaluated schools) vs those at remaining schools (remaining 75, remaining 65, and private schools) using a χ^2^ test. For details on classifications of schools, see eMethods 2 in Supplement 1.

## Discussion

This cross-sectional study found an absence of female medical leaders in Japan and a lack of diversity among high-ranking leaders in Japanese medical schools. Although the proportion of women physicians is increasing annually and governmental efforts toward gender equity are ongoing, women have yet to attain medical leadership positions in Japan.^2,3^ This is not exclusive to Japan; in the US, 11% to 13% of medical school deans are women.^4^ The median postgraduate experience of 38 years for high-ranking Japanese leaders corresponds to an age of approximately 63 to 64 years, aligning with previous studies indicating seniority and gender bias for medical society presidents.^2^ Excessive seniority bias impedes the advancement of competent young leaders and immobilizes the organization’s decision-making process.^5^ Moreover, the scarcity of high-ranking leaders holding degrees in social medicine (eg, business administration, public health, medical education, quality of care, and patient safety) warrants reflection. Lastly, the trend among Japanese medical leaders to remain in the institution where they graduated until retirement must be addressed given that a pronounced inclination toward this practice is detrimental to the discipline and likely restricts the dissemination of ideas, expertise, research, and teaching methods to society.^6^ This study has several limitations, including the inability to demonstrate temporal changes, characteristics associated with university hospital directors and medical school deans or their causal relationships, and the impact of the health care system in a sociocultural context, which may mean our results are not generalizable to other countries.

## Notes

Supplement 1.
eMethods 1.

eMethods 2.

Supplement 2.
Data Sharing Statement
