# Supplementary material for: Characteristics of Medical School Deans and University Hospital Directors in Japan
Source: JAMA Netw Open. 2024 Jan 11;7(1):e2351526. doi: 10.1001/jamanetworkopen.2023.51526 (PMC10784853; doi:10.1001/jamanetworkopen.2023.51526)
Supplement: Supplement 1. — eMethods 1. eMethods 2. [file jamanetwopen-e2351526-s001.pdf]

Supplemental Online Content

Watari T, Gupta A, Hayashi M, et al. Characteristics of medical school deans and university hospital directors in Japan. *JAMA Netw Open*. 2024;7(1):e2351526.
doi:10.1001/jamanetworkopen.2023.51526

eMethods 1.
eMethods 2.

This supplemental material has been provided by the authors to give readers additional information about their work.

© 2024 Watari T et al. *JAMA Network Open.*

eMethods 1.
Regarding individual data on the medical school deans and university hospital directors, we obtained medical license information (sex and year of registration) using the medical credentials search system of the Ministry of Health, Labor, and Welfare (Registered Doctor Search System, URL: https://licenseif.mhlw.go.jp/search_isei/jsp/top.jsp Accessed September 14, 2023). We further categorized the specialties of the directors and medical directors into clinical, basic experimental medicine and pathology, and social and public health based on their course and department websites. The new medical specialist board system classifies clinical fields into 19 primary specialty areas in Japan. Physicians who primarily engaged in basic research and education fields (eg, immunology, genetics, anatomy, physiology, forensic medicine) were categorized under the domain of basic medicine. The main research themes of the medical deans and directors were also classified into three categories (basic experimental medicine, clinical research, and social and public health) based on their research achievements published on the internet. Research fields were categorized based on publication fields (typically, ≥2/3 of all publications were within the same field). The three co-authors reviewed the classification of the major research categories. All but one had a medical license.

eMethods 2.
Each university medical school was classified as public (51 universities), including national and public universities and National Defense Medical College or private schools (31 universities). The imperial universities were founded by the Empire of Japan between 1886 and 1939 and run by the imperial government until the end of World War II. Therefore, it is considered a representation of the top seven public universities in Japan with a long history. The high-ranking schools were also identified using the historical classification of Japanese medical schools (17 high-ranking historical universities, including the seven aforementioned former imperial universities), consistent with Times Higher Education's world ranking (Times Higher Education World University Rankings. 2022. https://www.timeshighereducation.com/world-university-rankings/2022/world-ranking, Accessed September 23, 2023).

© 2024 Watari T et al. *JAMA Network Open.*
