# Supplementary material for: Characteristics of Medical School Deans and University Hospital Directors in Japan
Source: JAMA Netw Open. 2024 Jan 11;7(1):e2351526. doi: 10.1001/jamanetworkopen.2023.51526 (PMC10784853; doi:10.1001/jamanetworkopen.2023.51526)
Supplement: Supplement 2. — Data Sharing Statement [file jamanetwopen-e2351526-s002.pdf]

Data Sharing Statement

Watari. Characteristics of Medical School Deans and University Hospital Directors in Japan. *JAMA Netw Open*. Published online January 11, 2024. doi:10.1001/jamanetworkopen.2023.51526

Data
Data available: Yes
Data types: Deidentified participant data
How to access data: The data that support the findings of this study are available from the General Medicine Center, Shimane University; shimanegp@gmail.com (T.W), upon reasonable request.
When available: With publication

Supporting Documents
Document types: Statistical/analytic code
How to access documents: The data that support the findings of this study are available from the General Medicine Center, Shimane University; shimanegp@gmail.com (T.W), upon reasonable request.
When available: With publication

Additional Information
Who can access the data: researchers whose proposed use of the data has been approved
Types of analyses: for a specified purpose
Mechanisms of data availability: with investigator support
